# Network relationship between cognitive function and quality of life in community-dwelling older adults: an observational study from Beijing

**DOI:** 10.3389/fpubh.2024.1516895

**Published:** 2025-02-07

**Authors:** Yitian Ye, Yichun Zhang, Jiaju Ren, Yanbo Zhu

**Affiliations:** ^1^School of Management, Beijing University of Chinese Medicine, Beijing, China; ^2^School of Traditional Chinese Medicine, Beijing University of Chinese Medicine, Beijing, China

**Keywords:** cognitive assessment, quality of life, community-dwelling older adults, network analysis, health promotion

## Abstract

**Objective:**

This observational study aims to comprehensively explore the network relationship and mechanism of action between cognitive function and quality of life among community-dwelling older adults aged 60 and above in Beijing.

**Methods:**

The research encompassed a cohort of 323 older adults individuals residing in the community in Beijing. Data were collected from January to July 2024 using convenience sampling. Cognitive function was evaluated using the Chinese adaptation of the Montreal Cognitive Assessment (MoCA), while quality of life was assessed through the Medical Outcomes Study 36-Item Short-Form Health Survey version 2 (SF-36v2). Cognitive assessment involved seven dimensions, namely visuospatial/executive function, naming, attention, language, abstraction, delayed recall, and orientation. Concurrently, quality of life was assessed across eight dimensions: physical functioning, role physical, bodily pain, general health, vitality, social functioning, role emotional, and mental health. Network analysis graphs were developed to investigate the interrelationships among variables, identify central nodes, and evaluate stability.

**Results:**

In the network, the dimensions of social functioning, role-physical, physical functioning, general health, vitality, orientation, and language showed high centrality strength. The dimensions of physical functioning, role-physical, language, and orientation exhibited strong closeness and betweenness. There were strong associations between role-physical and social functioning, physical functioning and role-physical, and vitality and mental health. The centrality stability coefficients for strength, closeness, and betweenness were 0.672, 0.672, and 0.285, respectively.

**Conclusion:**

The network structure was stable, indicating that dimensions such as social functioning, role-physical, and physical functioning play pivotal roles influencing cognitive function in community-dwelling older adults. Orientation and language were the most representative dimensions of cognitive function and may serve as important targets for interventions aimed at improving cognitive function and subsequently enhancing QoL. These findings provide practical guidance for community health interventions. Future research should explore causal relationships and develop scalable strategies to support aging populations.

## Introduction

Globally, the health issues of older adults have garnered increasing attention as the aging process accelerates, particularly concerning the impact of cognitive function on quality of life (QoL) (1). Cognitive decline affects the independence and daily living abilities of older adults, often leading to increased dependency, social isolation, and psychological stress, which subsequently result in a significant reduction in quality of life (2). Studies have shown a close relationship between different dimensions of cognitive function and multiple aspects of quality of life in older adults (3, 4). For example, cognitive decline limits activities of daily living, reduces social interactions, and decreases life satisfaction and overall well-being (5, 6). Declines in memory and executive function can directly impact daily independence, while language impairments may be closely related to social interactions and interpersonal relationships (7–9).

In China, rapid aging poses a significant challenge, with the population aged 60 years or above expected to reach 487 million by 2050 (10). The quality of life (QoL) of older adults, encompassing physical, mental, and social well-being, is a critical determinant of their ability to live independently and participate in community life. Cognitive decline often intensifies challenges in these areas, increasing demands on social and healthcare systems. Addressing these challenges is imperative for enhancing QoL and developing sustainable community health strategies. As one of China’s largest metropolitan areas, Beijing is already facing substantial social and healthcare pressures resulting from this demographic shift. Addressing these issues through targeted interventions is essential for enhancing QoL and ensuring sustainable community health strategies. Understanding how cognitive ability affects the quality of life of older adults in community settings is crucial for designing effective community health strategies.

Previous research in China has shown that physical function, mental health, and social interactions are significantly affected by cognitive decline (11–13). However, most of these studies focus on singular dimensions and lack comprehensive analysis of the interplay between cognitive and quality of life (QoL) dimensions. Recent surveys have highlighted the prevalence of mild cognitive impairment (MCI) among community-dwelling older adults, with estimates 21.5% in urban regions like Beijing (14). Such findings underline the urgent need for a detailed examination of cognitive function and QoL dimensions specific to Beijing’s older population, facilitating targeted interventions in community settings.

Network analysis offers a novel and robust approach for elucidating these complex relationships (15). By conceptualizing the dimensions of cognitive function and quality of life as nodes within a network, we can systematically analyze their interconnections and identify the most influential features. These central features not only exhibit higher centrality within the network but also represent strategic intervention targets capable of exerting widespread influence across the entire system. This study examines the interconnections between cognitive function and quality of life (QoL) in community-dwelling older adults aged 60 and above in Beijing using network analysis. By identifying key factors such as language and orientation, it offers practical targets for community-level interventions and provides evidence-based recommendations for resource allocation and policy-making to support aging populations in urban settings.

## Methods

### Sample data

Data were collected using cross-sectional, onsite survey questionnaires from four community centers in Beijing between January 11 and July 19, 2024, through convenience sampling. The inclusion criteria were: (1) age 60 or above; (2) residency in the community for more than 1 year; (3) clear consciousness and the ability to express oneself verbally; (4) unimpaired activities of daily living; and (5) voluntary participation. The exclusion criteria included: (1) a diagnosis of dementia or severe cognitive impairment due to other causes (e.g., vascular dementia, brain injury, epilepsy); (2) severe mental disorders that prevent normal communication; (3) severe visual or hearing impairments that hinder communication; (4) severe allergies; and (5) noncompliance with the survey requirements.

The survey was implemented by a research team comprising postgraduate students and certified healthcare professionals, all of whom underwent rigorous, standardized training on cognitive and health assessment protocols prior to the study. Quality assurance was maintained through close supervision by senior researchers, periodic team evaluations, and a pilot survey conducted to refine the survey instruments and procedures.

The questionnaire comprised general information (e.g., height, weight, age, education level), cognitive assessments, and quality of life evaluations. Cognitive assessments were conducted collaboratively by participants and investigators, while the other sections were completed independently by participants. A total of 352 questionnaires were distributed onsite during community visits, with 340 completed questionnaires collected immediately, yielding a response rate of 96.6%. Among these, 323 valid questionnaires were retained for analysis. To enhance data accuracy, all questionnaire data were independently double-entered by two data entry personnel using EpiData version 3.1. Minimal missing data were identified and addressed using multiple imputation methods to ensure the robustness of the statistical analyses. Any non-inferable missing data and discrepancies in responses were resolved through follow-up interviews with the participants. Informed consent was obtained from all participants prior to the study.

### Measurement tools

#### Montreal cognitive assessment

The MoCA is a widely used tool for cognitive screening, developed by Nasreddine et al. in 2005 (16), aiming to rapidly assess mild cognitive impairment (MCI) and early cognitive decline. MoCA is particularly suitable for older adults and can be used in clinical and research settings to detect various dimensions of cognitive function. The MoCA includes seven domains, totaling 30 points:

1. **Visuospatial/Executive Function (0–5 points)**: Assesses abilities in copying figures, connecting paths, and cube drawing tasks.
2. **Naming (0–3 points)**: Assesses language ability through a picture-naming task.
3. **Attention (0–6 points)**: Includes forward and backward digit spans, arithmetic, and target search tasks.
4. **Language (0–3 points)**: Evaluates language fluency and expressive ability through sentence repetition and fluency tasks.
5. **Abstraction (0–2 points)**: Assesses abstract reasoning ability through two analogy tasks.
6. **Delayed Recall (0–5 points)**: Assesses short-term memory by asking participants to recall previously presented words.
7. **Orientation (0–6 points)**: Tests orientation to time and space.

The Chinese version of the MoCA used in this study has shown good validity and reliability in various regions and populations across China for screening mild cognitive impairment and early Alzheimer’s disease (17). The internal consistency of the sample in this study was assessed with a Cronbach’s *α* coefficient of 0.722.

### Medical outcomes study 36-item short-form health survey v2

The SF-36v2 (18) consists of 36 items covering eight dimensions: Physical Functioning (PF), Role Physical (RP), Bodily Pain (BP), General Health (GH), Vitality (VT), Social Functioning (SF), Role Emotional (RE), and Mental Health (MH). Scores for each dimension are first computed based on item weights, then transformed into standardized scores (0–100), with higher scores indicating better quality of life. The eight transformed scores of the SF-36v2, which has demonstrated good validity and reliability (19), were used as study variables. The internal consistency of the sample in this study was assessed with a Cronbach’s *α* coefficient of 0.880, supporting its use in assessing health-related quality of life.

### Data analysis

We utilized the qgraph (20) and bootnet (21) packages in R (version 4.2.1) to perform network analysis, using dimension scores from the Montreal Cognitive Assessment (MoCA) and the Medical Outcomes Study 36-Item Short-Form Health Survey version 2 (SF-36v2) as variables. A comprehensive description is provided detailing the construction of the adaptive correlation matrix and the calculation of centrality indices, including strength, closeness, and betweenness. Furthermore, the application of bootstrap methods is elaborated to assess the precision and robustness of network estimates, covering the generation of bootstrap confidence intervals and the computation of centrality stability coefficients.

An adaptive correlation matrix was utilized to compute centrality indices. In the network model, each variable was represented as a node, while the relationships between these variables were depicted as edges. Specifically, the seven dimensions of MoCA and the eight dimensions of SF-36 were modeled as nodes, with the correlations among these nodes represented as edges. The weight of each edge quantified the strength of the correlation between nodes, and edges could indicate either positive or negative associations, typically distinguished by color (e.g., green for positive associations and red for negative associations). The thickness of an edge represented the magnitude of the association.

To evaluate the importance of nodes within the network, three centrality indices were employed (22): strength, closeness, and betweenness. Strength is defined as the sum of the weights of all edges directly connected to a given node, indicating the intensity of its direct connections. Closeness is calculated as the inverse of the sum of the shortest path distances from a node to all other nodes; a higher value indicates a greater ability of the node to quickly influence other nodes. Betweenness measures how frequently a node lies on the shortest path between two other nodes, thereby assessing its role as a bridge within the network. These indices are typically expressed as standardized z-scores, with higher values denoting greater importance within the network. The spatial distance between nodes in the correlation graph is also meaningful; shorter distances indicate stronger relationships between nodes.

To further assess the accuracy and stability of the “cognition-quality of life” network, we set the number of bootstrap samples to 1,000 and applied the bootstrapping method to evaluate the precision and robustness of network estimates (23). First, the accuracy of the estimates was evaluated by constructing bootstrap confidence intervals (CIs) for each edge weight. The greater the overlap between the CIs of edge weights, the lower the ability of the graphical representation to distinguish differences between these weights. Second, the stability of the centrality indices was assessed by bootstrapping resampling of sample subsets. Specifically, we examined how the centrality stability coefficient (CS-coefficient) changed as the proportion of the sample subset decreased (e.g., comparing 100% of the sample to 30%). The CS-coefficient represents the maximum proportion of data points that can be removed while still maintaining a correlation of at least 0.7 between the original network statistics and those calculated with the reduced sample. This coefficient should not be below 0.25 and ideally should exceed 0.5 (21). The faster the centrality indices change with a reduced sample, the lower their stability. Finally, bootstrap analyses were performed on paired differences in edge weights and node centralities to determine whether there were statistically significant differences between edges or nodes.

## Results

### Descriptive statistics of variables

The general characteristics of the sample and the mean, standard deviation, skewness, and kurtosis of the variables included in the network analysis are shown in Table 1. Among the sample, there were 133 males (40.06%), and the mean age was (72.15 ± 8.41) years. In the cognitive assessment, the dimensions of naming and orientation showed high kurtosis, while in the QoL evaluation, the role-emotional dimension had the highest mean score, and general health had the lowest mean score, reflecting the characteristics of the participants.

**Table 1:** Status of sample and variables in network analysis.

Sample characteristics	*N* = 323	Range
Male, n (%)	133 (40.06%)	
Age, mean (SD)	72.15 ± 8.41	60–96
With spouse, n (%)	288 (89.16%)	
Years of education, mean (SD)	11.76 (3.94)	0–25
Education level, n (%)		
No formal	8 (2.48%)	
Primary	30 (9.29%)	
Secondary and above	285 (88.23%)	

Variable symptoms	Label	Mean	SD	Max	Min	Range	Skewness	Kurtosis
Visuospatial/Executive	MoCA1	3.63	1.31	5	0	0–5	−0.86	0.23
Naming	MoCA2	2.85	0.49	3	0	0–3	−3.92	17.19
Attention	MoCA3	5.41	1.01	6	0	0–6	−2.47	8.15
Language	MoCA4	2.57	0.83	3	0	0–3	−1.94	2.78
Abstraction	MoCA5	1.76	0.51	2	0	0–2	−2.03	3.30
Delayed Recall	MoCA6	3.09	1.53	5	0	0–5	−0.38	−0.76
Orientation	MoCA7	5.77	0.81	6	0	0–6	−5.18	31.38
Physical Functioning	PF	78.79	25.81	100	0	0–100	−1.71	2.20
Role−Physical	RP	80.73	24.76	100	0	0–100	−1.31	0.78
Bodily Pain	BP	74.24	22.27	100	21	0–100	−0.40	−0.76
General Healthy	GH	61.16	22.83	100	0	0–100	−0.42	−0.40
Vitality	VT	75.89	17.22	100	25	0–100	−0.57	−0.26
Social Functioning	SF	82.12	21.95	100	12.5	0–100	−1.42	1.57
Role−Emotional	RE	89.94	17.86	100	16.67	0–100	−2.15	4.54
Mental Health	MH	84.75	14.03	100	25	0–100	−1.30	1.90

### Network structure analysis

**Figure 1:**
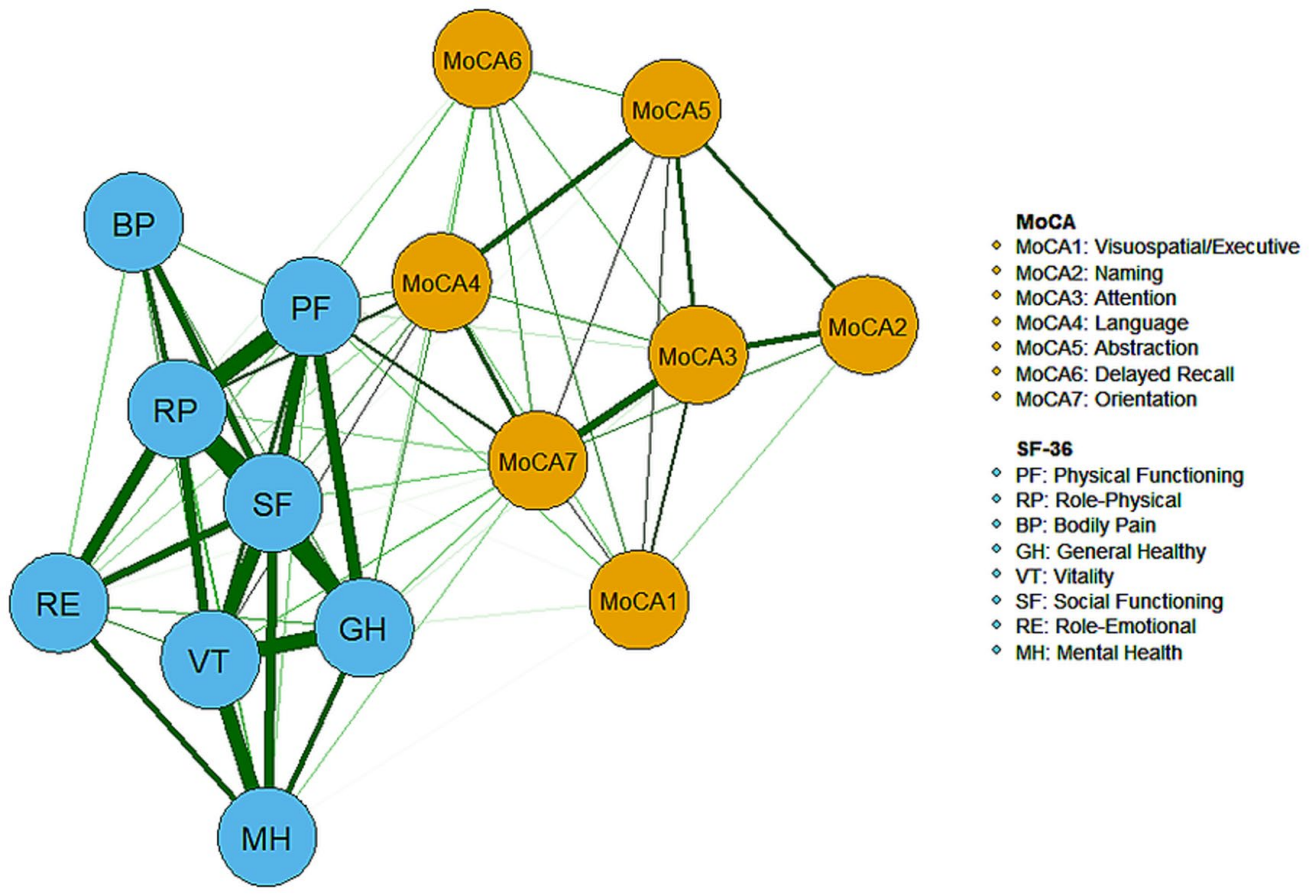
The network of cognition and quality of life dimensions.

The network structure of cognitive and QoL dimensions among community-dwelling older adults in Beijing is depicted in Figure 1, where all correlations between dimensions are positive (represented by green edges). Within QoL, the dimensions of role-physical, social functioning, physical functioning, and general health were closely interconnected (indicated by thicker edges) and held more central positions compared to other dimensions, reflecting their strong explanatory power and high correlations in the QoL evaluation. Additionally, strong correlations were observed between the role-physical and role-emotional dimensions, as well as between vitality and the dimensions of mental health, social functioning, general health, and role-physical.

**Figure 2:**
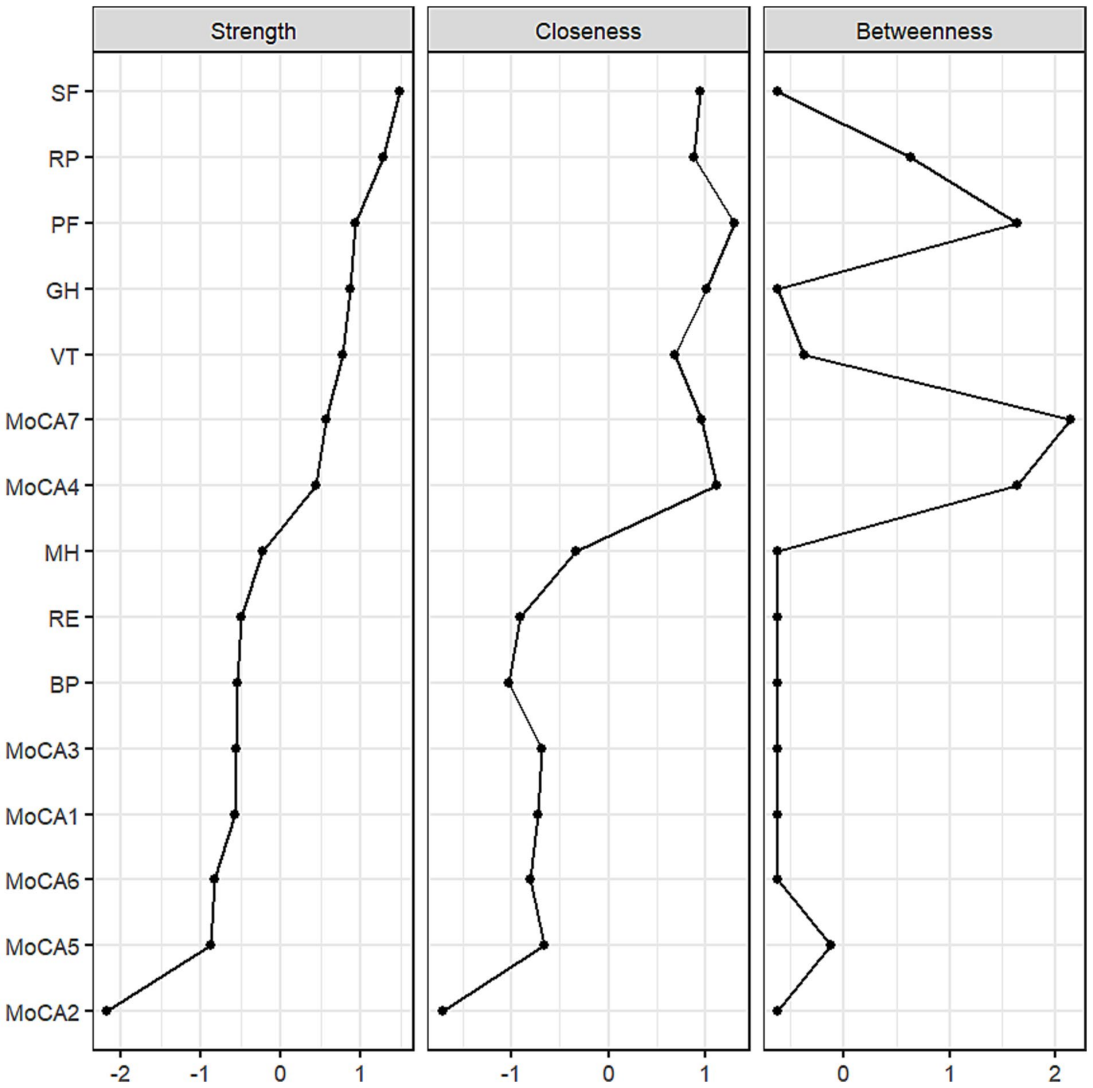
The centrality indices of the network.

In terms of cognition, strong correlations were found between the dimensions of attention and orientation, as well as between naming and attention. The central positioning of the language and orientation dimensions highlights their prominence in the network and their strong association with QoL. The centrality of both cognitive and QoL dimensions within the network is further illustrated in Figure 2. The dimensions of social functioning, role-physical, physical functioning, general health, vitality, language, and orientation exhibited high strength, while physical functioning, role-physical, language, and orientation demonstrated higher closeness and betweenness.

### Network accuracy and stability analysis

**Figure 3:**
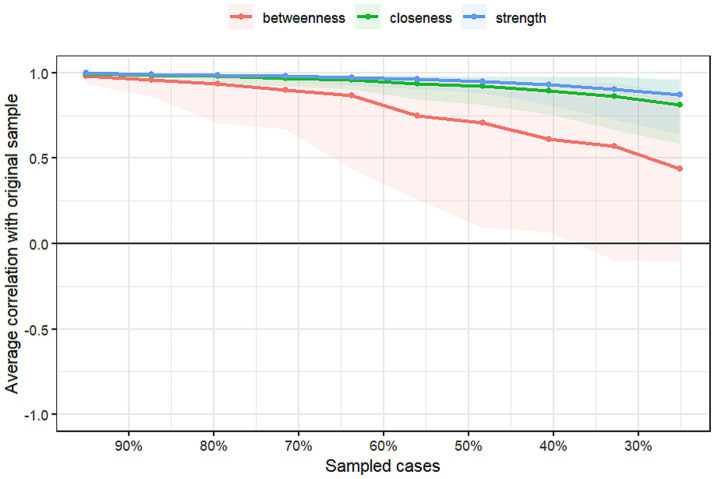
Centrality stability of the network.

**Figure 4:**
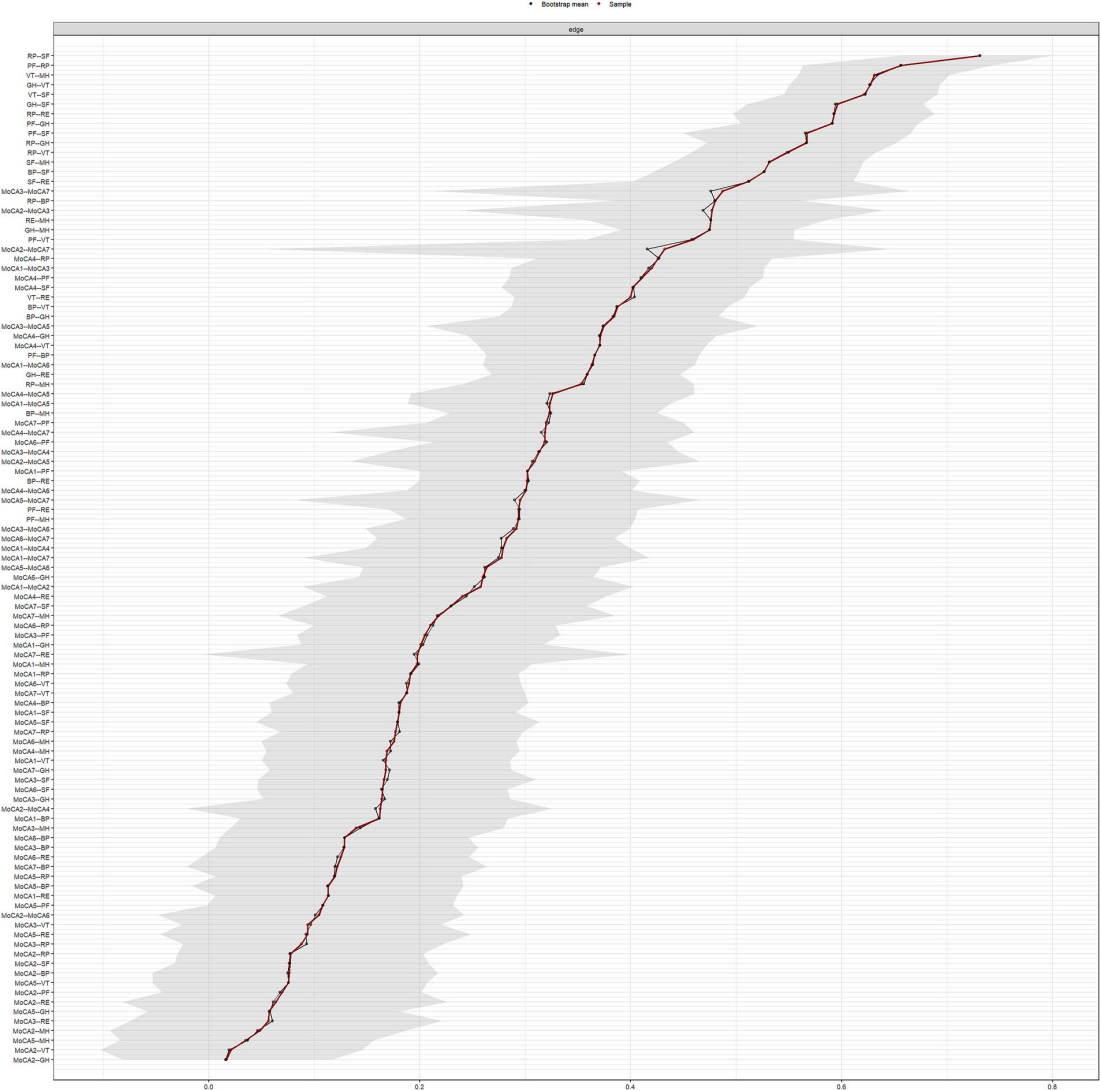
Edge-weight Bootstrapped CIs of the network.

**Figure 5:**
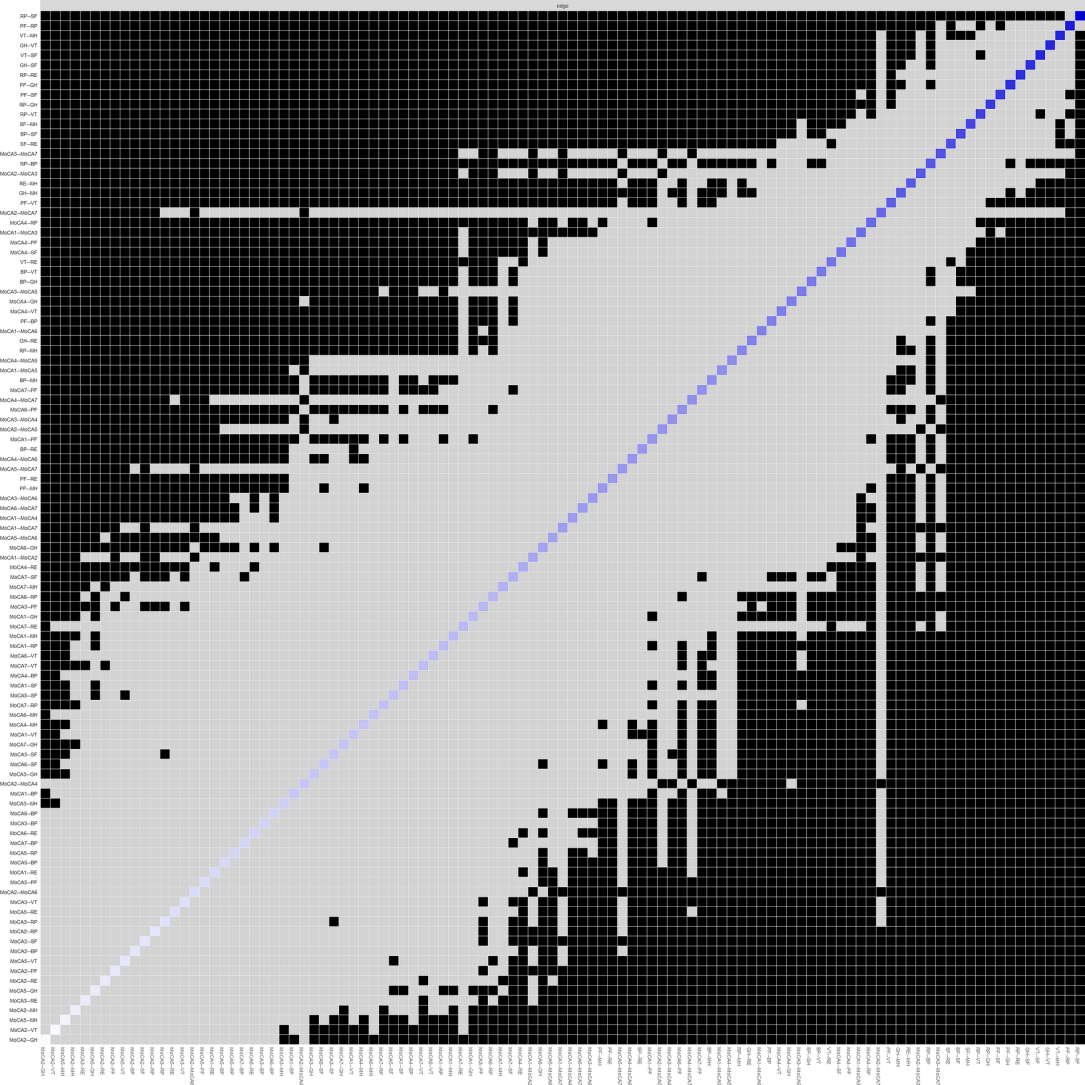
Edge differences of the network.

**Figure 6:**
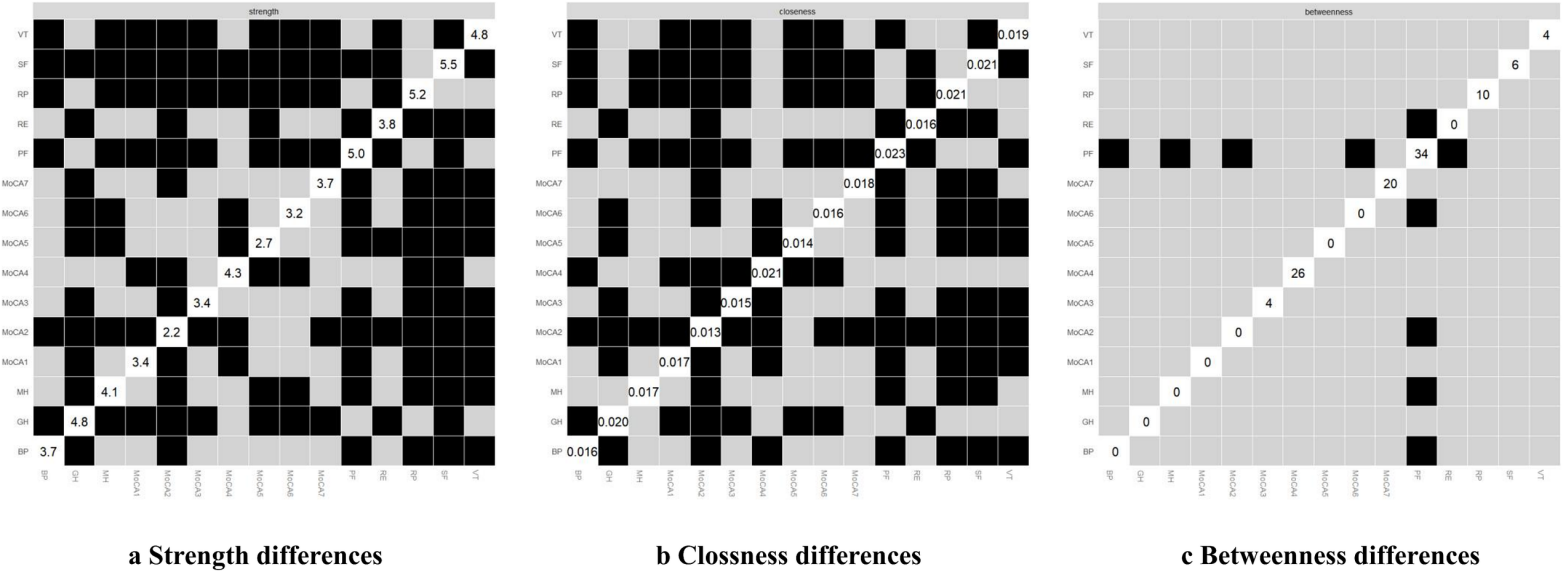
Centrality differences of the network. In figure **(a-c)**, white squares represent the value of node strength, clossness and betweenness respectively. The y-axis and x-axis correspond to each node. Black squares indicate nodes with significant differences, while grey squares denote non-significant differences.

The results regarding the accuracy and stability of the network structure are presented in Figures 3–6. Figure 3 illustrates the stability of the centrality indices, demonstrating that as the proportion of resampled data decreases, the stability of strength, closeness, and betweenness centrality remains consistent. The calculated CS-coefficients for strength, closeness, and betweenness are 0.672, 0.672, and 0.285, respectively, highlighting that the network’s centrality measures are relatively stable. Figure 4 shows the associations among the network’s 105 edges, where the confidence intervals for highly correlated edges generally do not cross zero, demonstrating that the edge structure is stable based on point estimates.

As shown in Figure 5, dark squares predominantly cover the relationships within the quality of life dimensions and between the quality of life and cognitive dimensions, while gray squares are mostly found between cognitive dimensions. Together with Figure 1, these findings indicate that significant differences are more prevalent between QoL dimensions and between QoL and cognitive dimensions, while fewer notable differences within the cognitive dimensions themselves. Among the cognitive dimensions depicted in Figure 4, the strongest correlations in descending order are between attention and orientation, naming and attention, and naming and orientation. The confidence intervals for “naming and orientation” display greater variability in Figure 4, and fewer significant differences with other edges are found in Figure 5 (indicated by the presence of more gray squares). This suggests that attention and orientation, as well as naming and attention, demonstrate moderate correlations within the cognitive domain. For the edges connecting cognitive and QoL dimensions in Figure 4, those involving the language or orientation dimensions frequently show stronger correlations, demonstrating a robust association between these dimensions and QoL. Considering their high centrality indices, we can infer that the orientation and language dimensions are key representatives of cognitive function and have a substantial impact on QoL.

The differences in centrality are presented in Figures 6a–c. In the network, differences in strength and closeness centrality are more pronounced than those in betweenness centrality (Figures 6a,b exhibit more dark squares compared to Figure 6c). In terms of strength and closeness centrality differences (Figures 6a,b), social functioning, role-physical, physical functioning, general health, vitality, and naming dimensions display more dark squares in the sample network. Combined with the magnitude of strength and closeness (Figure 2), this indicates that these dimensions have significantly higher strength and closeness compared to others. Regarding betweenness centrality differences (Figure 6c), physical functioning shows more dark squares in the sample network, and based on its betweenness magnitude (Figure 2), it can be concluded that physical functioning has significantly higher betweenness than other dimensions. These results collectively support the accuracy and stability of the network structure as depicted in Figure 1.

## Discussion

In this study, we utilized network analysis to explore the relationship between cognitive function and quality of life among community-dwelling older adults in Beijing. Our findings demonstrate that dimensions such as social functioning, role-physical, and physical functioning exhibited high centrality and closeness within the network structure, highlighting their critical role in linking quality of life with cognitive abilities in older adults.

The strong associations observed between physical functioning, role-physical, and other quality of life dimensions are consistent with findings from prior studies, which underscores the positive impact of robust physical health and sufficient social support on overall well-being in older adults (24–26). The close relationship between role-physical and social functioning further emphasizes the importance of maintaining good physical health to sustain effective social relationships among older adults. This is consistent with the findings of Kelly et al. (6), who demonstrated that social engagement and social support are essential for enhancing cognitive function and quality of life in older adults individuals.

Furthermore, our findings showed that the language function dimension held high centrality within the network, suggesting that language may be a key indicator of cognitive function and a core dimension influencing quality of life. This observation aligns with Woodward’s findings (27), which indicated that language impairment is significantly negatively associated with daily living abilities and social engagement in patients with Alzheimer’s disease, thereby corroborating the present study’s findings.

In addition to language, the orientation dimension also showed a significant association with quality of life in the network. The high centrality of the orientation dimension emphasizes its pivotal role in both cognitive function and quality of life. Maintaining accurate temporal and spatial orientation is crucial for the self-care abilities and perceived well-being of older adults (28). Declines in both language abilities and spatial orientation have a direct impact on the mental health of older adults, which is consistent with the findings by Yang et al. (29), who also elaborated that maintaining temporal and spatial orientation is associated with better self-care capacity and subjective health among the older adults.

The favorable results of the network stability analysis further validate the robustness of this network, suggesting that improving social functioning, role-physical, and physical functioning could contribute positively to enhancing the quality of life in older adults individuals. Within the cognitive domain, the high centrality of nodes related to language and orientation, along with the strong correlations between these dimensions, underscores the notion that training in language and orientation may be an effective strategy to enhance cognitive function and thereby improve quality of life in older adults (30).

A deeper exploration of the interactions between dimensions such as physical functioning, social functioning, vitality, and language is necessary to fully understand how these factors collectively influence cognitive functioning and quality of life. While these dimensions are often studied in isolation, their dynamic interplay likely holds the key to more effective interventions and a comprehensive understanding of aging. For example, improving physical health might enable older adults to engage more fully in social activities, which in turn can have a positive feedback effect on their physical capabilities by fostering a sense of purpose and social connection. This reciprocal relationship, however, is underexplored and could be crucial in understanding how to simultaneously enhance both physical and cognitive functions. Moreover, the boundaries between certain dimensions, such as vitality and general health, appear to be more fluid than originally conceived. Vitality is often regarded as a purely physical state, yet it also has profound psychological implications. Peng et al. (12) suggest that vitality can act as a buffer, mediating the relationship between physical frailty and mental health, thereby demonstrating how psychological and physical well-being are intertwined. This highlights the importance of developing interventions that go beyond improving physical health alone, recognizing that vitality plays a crucial role in bridging physical and mental well-being. Enhancing vitality, for example, through targeted physical activity programs, can not only improve physical function but also alleviate symptoms of depression, creating a more balanced approach to aging (31). Language is not just a means of communication but a critical tool for navigating social contexts, which could directly impact an individual’s spatial and temporal orientation. A better understanding of how these two cognitive functions influence each other could reveal new avenues for intervention (32), where improving one dimension could enhance the other, leading to a more holistic improvement in quality of life.

Unlike prior studies that focused on correlation analysis, comparative analysis, or structural equation modeling, this study adopted a network analysis approach, offering a new perspective for understanding the interaction between cognitive function and quality of life (33–35). An important advantage of network analysis lies in its ability to reveal both direct and indirect relationships between variables and to identify key nodes, facilitating the design of targeted intervention strategies. Nodes with high centrality in the network typically have greater intervention potential, and targeted interventions for these nodes may have far-reaching effects across the entire network (36). Our findings, in comparison with those from previous studies, support this view, suggesting that focusing on improving social functioning, physical functioning, role-physical, language, and orientation can have a positive impact on cognitive levels and quality of life in older adults. Additionally, our results illustrate the bidirectional relationship between cognition and quality of life, revealing complex interaction mechanisms between these two domains through network structure analysis, particularly in how enhancing specific cognitive abilities can improve the overall quality of life in older populations. These findings are crucial for developing health intervention programs for older adults. Public health practitioners can develop more targeted health promotion strategies by strengthening these core functions, which may help older adults maintain robust social connections and physical well-being, ultimately improving their overall quality of life.

Despite the valuable insights gained in understanding the relationship between cognitive function and quality of life in older adults, several limitations of this study should be acknowledged. First, as a cross-sectional observational study, it does not allow for causal inferences between variables or an examination of how the “cognition-quality of life” network changes over time or under various intervention conditions. Future studies should consider using longitudinal designs and controlled interventions to explore causal relationships between cognition and quality of life. Second, the sample for this study was limited to community-dwelling older adults in Beijing, which may affect the generalizability of the findings (e.g., older adults in economically advanced regions may generally exhibit higher cognitive levels) (37). Expanding the sample to include older adults individuals from different countries, regions, and cultural backgrounds is necessary to enhance the external validity of the findings. Furthermore, while this study accounted for some potential confounding factors (e.g., chronic illnesses, medication use) during data collection, further subgroup analyses controlling for potential confounders would help improve the accuracy of the results.

## Conclusion

This study highlights the critical interplay between cognitive function and quality of life in community-dwelling older adults. Core dimensions, including social functioning, role-physical, physical functioning, language, and orientation emerged as central within the network, offering actionable targets for intervention. The findings emphasize the utility of network analysis in understanding complex multidimensional relationships and designing targeted strategies to enhance the well-being of aging populations. Future research should prioritize longitudinal designs, diverse samples, and advanced modeling techniques to build upon these findings and inform effective public health interventions. By focusing on these core dimensions, practitioners can develop comprehensive strategies that simultaneously address cognitive health and quality of life, fostering healthier aging populations.

## Data Availability

The data analyzed in this study is subject to the following licenses/restrictions: all data are derived from the self-established databases. The datasets used and/or analyzed during the current study are available from the corresponding author upon reasonable request. Requests to access these datasets should be directed to Yanbo Zhu, yanbo0722@sina.com.
